# Supplementary material for: Cryo-EM structure of transmembrane AAA+ protease FtsH in the ADP state
Source: Commun Biol. 2022 Mar 23;5:257. doi: 10.1038/s42003-022-03213-2 (PMC8943139; doi:10.1038/s42003-022-03213-2)
Supplement: Supplementary file 2 — Supplemental information [file 42003_2022_3213_MOESM2_ESM.pdf]

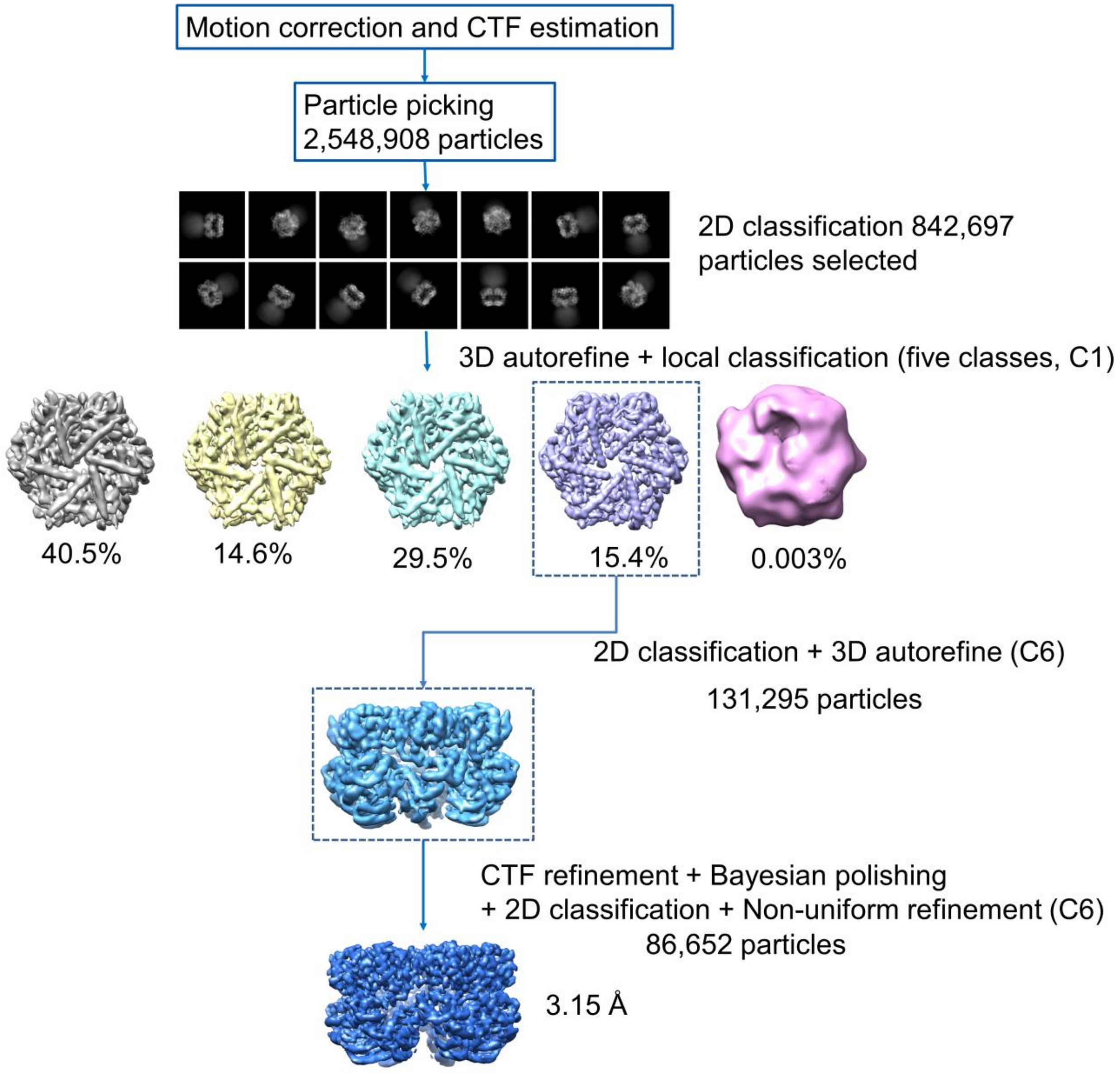

Supplementary Figure 1. A data analysis workflow for the reconstruction of ADP-state *Tm*FtsH.

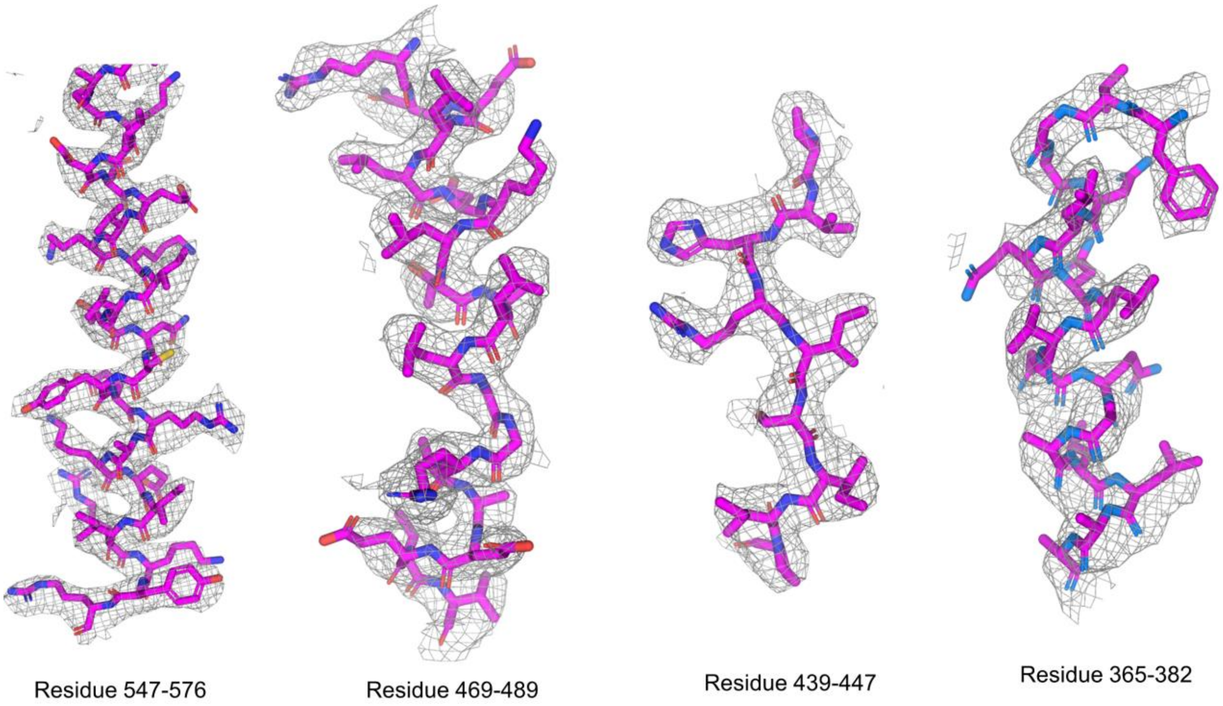

Supplementary Figure 2. Cryo-EM densities for the regions in *Tm*FtsH to show the quality of map.

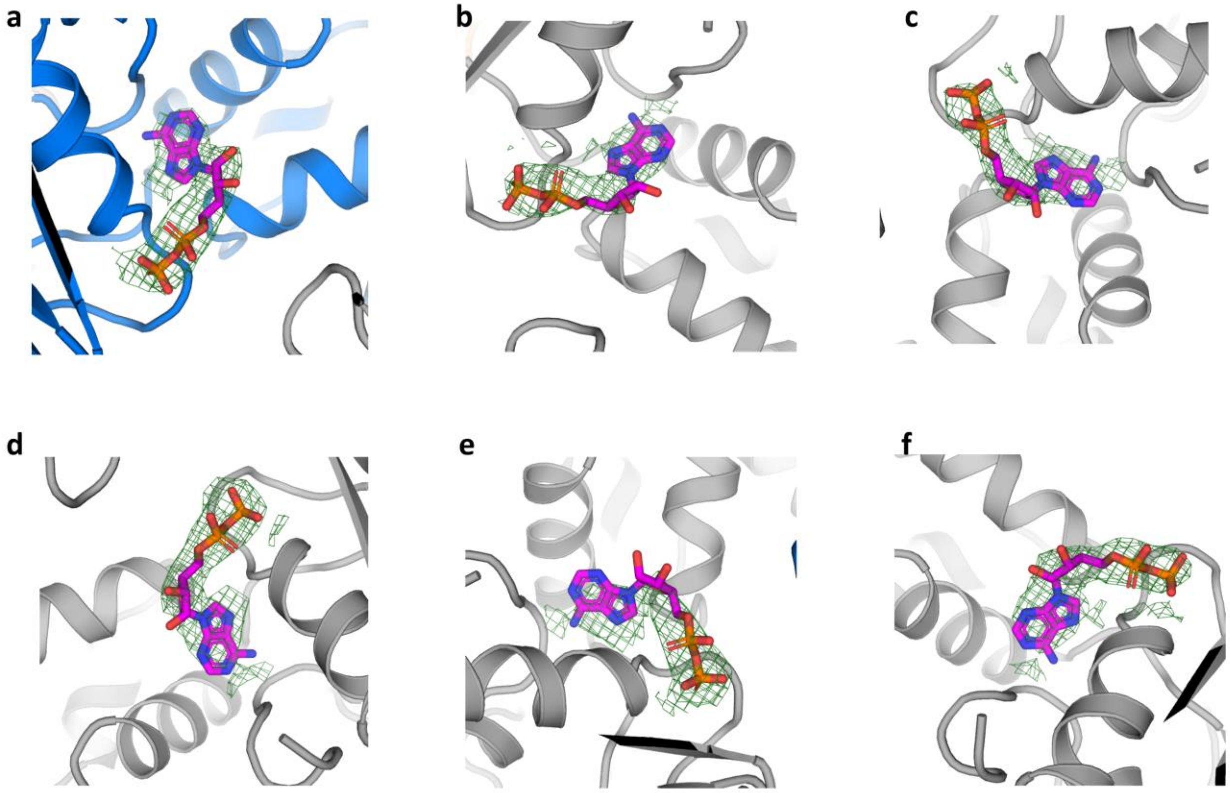

Supplementary Figure 3. Cryo-EM densities for the six ADP molecules with the structure reconstructed without using a symmetry. a-f Cryo-EM densities for ADP molecules shown as green isomeshes.

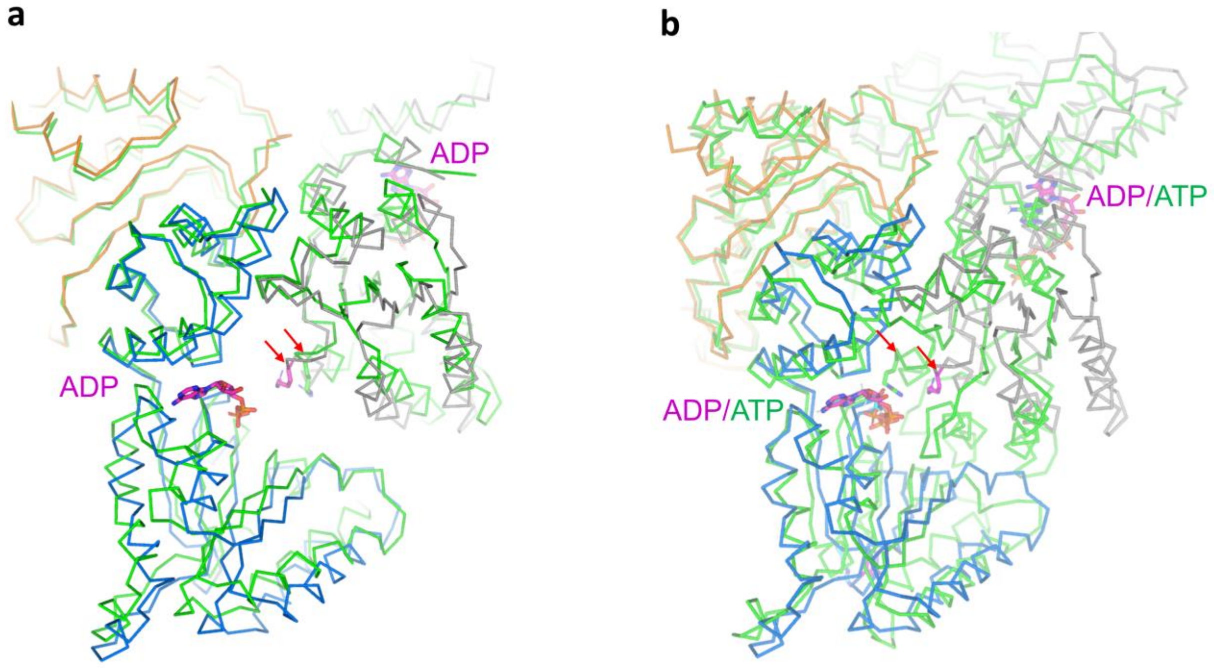

Supplementary Figure 4. Structural comparisons of ADP-state structure with the apo- and ATP-state structures. a Superimposition of ADP-state structure with the apo-state structure (PDB code 3KDS). One ADP-state subunit was colored orange for protease and marine for ATPase. The next clockwise subunit was colored in gray. The apo-state structure was colored in green. ADP molecules were shown as sticks. Red arrows indicate the Cα positions of Arg finger. **b** Superimposition of ADP-state structure with the ATP-state structure (PDB code 6AZ0). One ADP-state subunit was colored orange for protease and marine for ATPase, and the next clockwise subunit was colored in gray. The ATP-state structure was colored in green. ADP and ATP molecules were shown as sticks and colored differently. Red arrows indicate the Cα positions of Arg finger.
