# Supplementary material for: Cryo-EM structure of transmembrane AAA+ protease FtsH in the ADP state
Source: Commun Biol. 2022 Mar 23;5:257. doi: 10.1038/s42003-022-03213-2 (PMC8943139; doi:10.1038/s42003-022-03213-2)
Supplement: Supplementary file 3 — Description of Additional Supplementary Files [file 42003_2022_3213_MOESM3_ESM.pdf]

Description of Additional Supplementary Files

File name: Supplementary Data 1
Description: Source data for Fig. 1c.
